# Anatomy and Taxonomic Status of the Chasmosaurine Ceratopsid *Nedoceratops hatcheri* from the Upper Cretaceous Lance Formation of Wyoming, U.S.A

**DOI:** 10.1371/journal.pone.0016196

**Published:** 2011-01-20

**Authors:** Andrew A. Farke

**Affiliations:** Raymond M. Alf Museum of Paleontology, Claremont, California, United States of America; College of the Holy Cross, United States of America

## Abstract

**Background:**

The validity of *Nedoceratops hatcheri*, a chasmosaurine ceratopsid dinosaur known from a single skull recovered in the Lance Formation of eastern Wyoming, U.S.A., has been debated for over a century. Some have argued that the taxon is an aberrant Triceratops, and most recently it was proposed that *N. hatcheri* represents an intermediate ontogenetic stage between “young adult” and “old adult” forms of a single taxon previously split into *Triceratops* and *Torosaurus*.

**Methodology/Principal Findings:**

The holotype skull of *Nedoceratops hatcheri* was reexamined in order to map reconstructed areas and compare the specimen with other ceratopsids. Although squamosal fenestrae are almost certainly not of taxonomic significance, some other features are unique to *N. hatcheri*. These include a nasal lacking a recognizable horn, nearly vertical postorbital horncores, and relatively small parietal fenestrae. Thus, *N. hatcheri* is tentatively considered valid, and closely related to *Triceratops* spp. The holotype of *N. hatcheri* probably represents an “old adult,” based upon bone surface texture and the shape of the horns and epiossifications on the frill. In this study, *Torosaurus* is maintained as a genus distinct from *Triceratops* and *Nedoceratops*. Synonymy of the three genera as ontogenetic stages of a single taxon would require cranial changes otherwise unknown in ceratopsids, including additions of ossifications to the frill and repeated alternation of bone surface texture between juvenile and adult morphotypes.

**Conclusions/Significance:**

*Triceratops*, *Torosaurus*, and likely *Nedoceratops*, are all distinct taxa, indicating that species richness for chasmosaurine ceratopsids in the Lance Formation just prior to the Cretaceous-Paleocene extinction was roughly equivalent to that earlier in the Cretaceous.

## Introduction

In 1889, John Bell Hatcher and his field crew undertook the first of four productive seasons of exploration in the late Maastrichtian-aged Lance Formation of Niobrara County, Wyoming, USA, under the auspices of the United States Geological Survey. These expeditions resulted in a massive collection of vertebrate fossils, ranging from fish to mammals to dinosaurs. Most remarkably, over 30 ceratopsian (horned dinosaur) skulls were recovered [1]. These included collections of fragments, disarticulated specimens, and complete, articulated crania, which were used to erect a number of genus- and species-level taxa that have literally defined our concept of “horned dinosaurs.” Among the initial series of discoveries were 12 species of *Triceratops* (later variably sunk into one or two species, *Triceratops horridus* and *Triceratops prorsus*; e.g., [2], [3]); *Sterrholophus flabellatus* (synonymous with *Triceratops horridus*; [3]); *Torosaurus latus* and *Torosaurus gladius* (only *T. latus* is now considered valid; [4]); and *Nedoceratops hatcheri* [5], [6].

**Figure 1:**
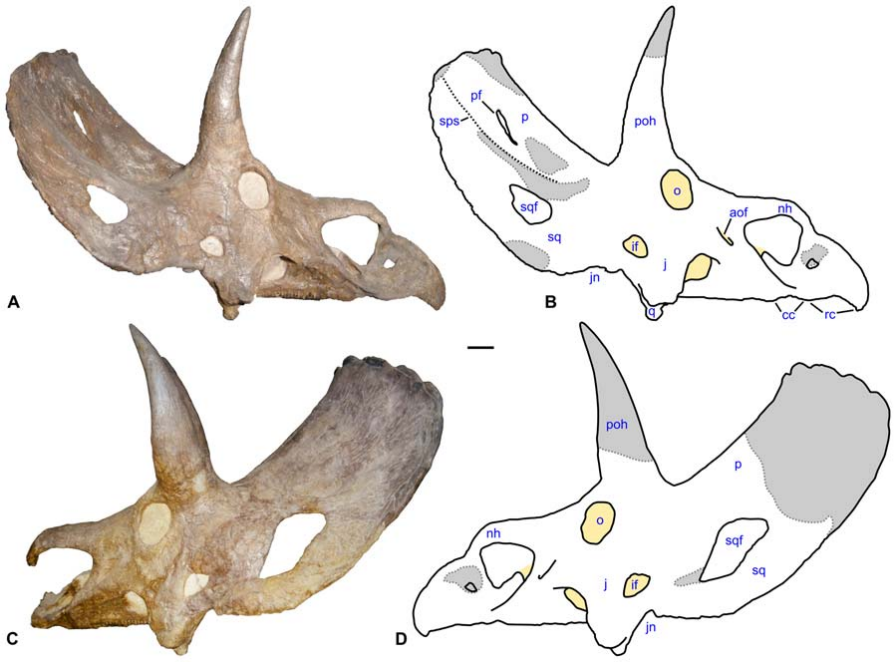
Skull of *Nedoceratops hatcheri*, USNM 2412. **A.** Photograph in right lateral view. **B.** Interpretive line drawing in right lateral view, with major reconstructed areas indicated in gray and matrix indicated in yellow. **C.** Photograph in left lateral view. **D.** Interpretive line drawing in left lateral view, with major reconstructed areas indicated in gray and matrix indicated in yellow. In **C**, the rostral end of the skull was broken away at the time of photography. **Abbreviations:** **aof**, antorbital fenestra; **cc**, caudal curve of oral margin; **if**, infratemporal fenestra; **j**, jugal; **jn**, jugal notch of squamosal; **nh**, nasal horncore; **o**, orbit; **p**, parietal; **pf**, parietal fenestra; **poh**, postorbital horncore; **q**, quadrate; **rc**, rostral curve of oral margin; **sps**, squamosal-parietal suture; **sq**, squamosal; **sqf**, squamosal fenestra. Scale bar equals 10 cm.

*Nedoceratops hatcheri*, a taxon erected based upon a single, nearly complete skull of a large chasmosaurine (∼1.8 m greatest skull length; Figures 1 – [Fig pone-0016196-g003] [Fig pone-0016196-g004] [Fig pone-0016196-g005] 6), has suffered a long history of proposed synonymy, incomplete description and figuring, and nomenclatural confusion. Hatcher originally intended to name the taxon as a new genus and species, but died before he could do so [7]. Thus, it fell upon Richard S. Lull to attach the name *Diceratops hatcheri* to a brief description (scarcely a full page of text) penned by Hatcher [5]. Decades later, Lull changed his mind on the generic status of *Diceratops* and relegated it to a subgenus of *Triceratops* [7]. In 1986, Ostrom and Wellnhofer [2] posited that *Diceratops hatcheri*, along with all of the named species of *Triceratops*, fell within the expected range of variation for a single taxon, *Triceratops horridus*. This situation remained until 1996, when Forster, in her revision of *Triceratops* and related forms, suggested that *Diceratops* was indeed a valid taxon [3]. This opinion has not been unanimously accepted among ceratopsid workers, with many considering the taxon to be synonymous with *Triceratops horridus* (e.g., [8], [9]). Most recently, Scannella and Horner [10] hypothesized that *Diceratops hatcheri* represented a transitional form between the “young adult” and “old adult” forms of *Triceratops* (with *Torosaurus* as the fully adult form of *Triceratops*).

**Figure 2 pone-0016196-g002:**
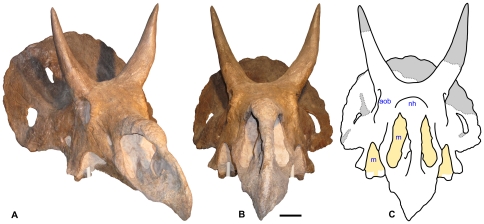
Skull of *Nedoceratops hatcheri*, USNM 2412. **A.** Photograph in right oblique view. **B.** Photograph in rostral view. **C.** Interpretive line drawing in rostral view, with major reconstructed areas indicated in gray and matrix indicated in yellow. **Abbreviations:** **aob**, antorbital buttress; **m**, matrix and metal supports; **nh**, nasal horncore. Scale bar equals 10 cm, but note that parallax prevents accurate measurement of parts of the skull caudal to the external naris.

**Figure 3 pone-0016196-g003:**
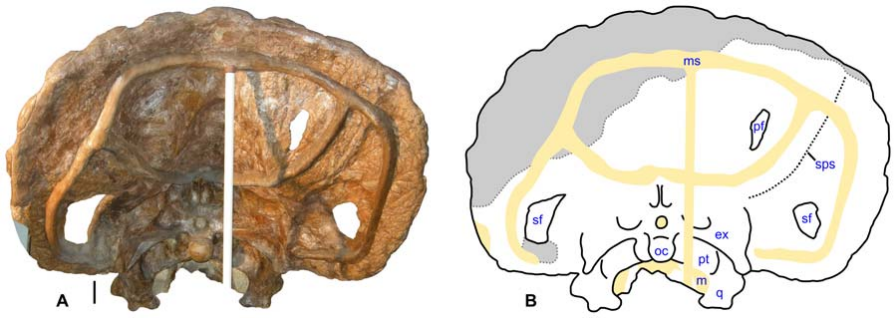
Skull of *Nedoceratops hatcheri*, USNM 2412. **A.** Photograph in caudal view. **B.** Interpretive line drawing in caudal view, with major reconstructed areas indicated in gray and matrix and metal supports indicated in yellow. **Abbreviations:** **ex**, exoccipital; **m**, matrix; **ms**, metal support; **q**, quadrate; **oc**, occipital condyle; **pf**, parietal fenestra; **pt**, pterygoid; **sf**, squamosal fenestra; **sps**, squamosal-parietal suture. Scale bar equals 10 cm.

**Figure 4 pone-0016196-g004:**
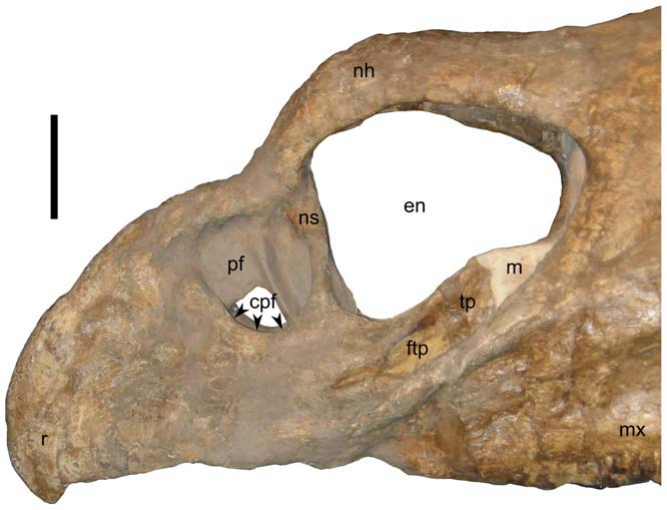
Close-up of rostral end of skull of *Nedoceratops hatcheri*, USNM 2412, in left lateral view. **Abbreviations: cpf**, canal at edge of premaxillary fossa; **en**, endonaris; **ftp**, fossa on triangular process; **m**, matrix; **mx**, maxilla; **nh**, nasal horncore; **ns**, narial strut; **pf**, premaxillary fossa; **r**, rostral bone; **tp**, triangular process. Scale bar equals 10 cm.

**Figure 5 pone-0016196-g005:**
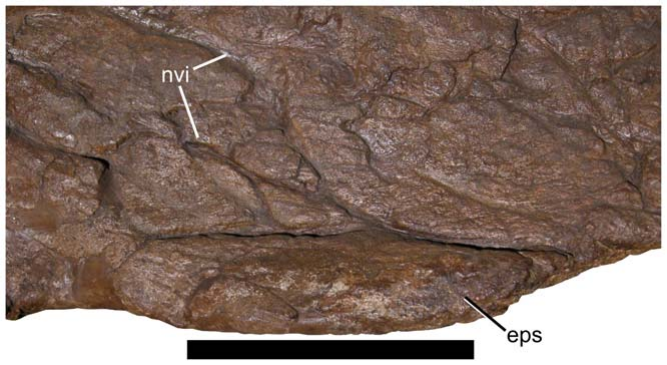
Close-up of episquamosal on left squamosal of *Nedoceratops hatcheri*, USNM 2412. Note the neurovascular impressions on the squamosal, suggestive of adult status. The caudal end is to the right of the image. **Abbreviations: eps**, episquamosal; **nvi**, neurovascular impressions. Scale bar equals 10 cm.

**Figure 6:**
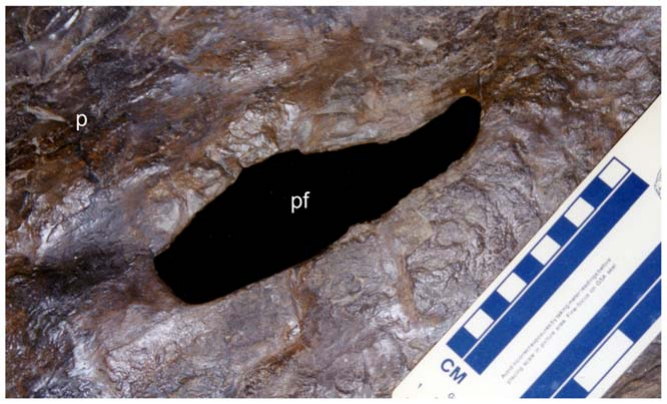
Close-up of right side of parietal and parietal fenestra of *Nedoceratops hatcheri*, USNM 2412. The bone is shown in dorsal view, with the caudal end to the left of the image. **Abbreviations:** **p**, parietal; **pf**, parietal fenestra. Scale bar equals 10 cm.

Finally, the name “*Diceratops*” itself has experienced a confusing history. After it was discovered that the genus name *Diceratops* was preoccupied by an extant wasp [11], two competing replacement names were proposed: *Nedoceratops* [6] and *Diceratus* [12]. Although initially less widely known, *Nedoceratops* was the first to be published and hence has priority as the generic name.

The type and only specimen for *Nedoceratops hatcheri*, USNM 2412, remains poorly understood. In particular, the skull preserves features such as small parietal fenestrae, paired squamosal fenestrae, and erect supraorbital horns (Figures 1–3), that have been variably considered individual variation, ontogenetic features, pathology or valid characters separating the taxon from *Triceratops* (e.g., [1]–[3], [5], [7], [10], [13]). This debate over the validity of *N. hatcheri* stems largely from inadequately brief and occasionally incorrect descriptions of the specimen, incomplete documentation of restoration on the skull, and differing opinions among ceratopsid workers. Although the last situation continues, this paper addresses the first two issues as a means to move the discussion forward. An understanding of the anatomy and taxonomic status of *N. hatcheri* is important for answering two questions in particular: 1) How many species of large ceratopsid co-existed in the area of the Lance and Hell Creek formations; and 2) If *N. hatcheri* is valid, what separates it from related forms such as *Triceratops* and *Torosaurus*?

Here, I present the first comprehensive description, diagnosis and illustration of the type specimen for *Nedoceratops hatcheri*. The degree of restoration of the skull is fully documented (Figures 1–3), considerably altering some previous interpretations of the cranial anatomy (e.g., [1], [7]). The holotype skull of *N. hatcheri* is compared to the morphologies in the coeval *Triceratops* and *Torosaurus* in particular, in an attempt to address the validity of *N. hatcheri*. Finally, I address the recently proposed hypothesis that *Triceratops* and *Torosaurus* are two stages of an ontogenetic series for a single taxon, with *Nedoceratops* possibly representing a transitional form between the two morphs [10].

### Institutional abbreviations

AMNH, American Museum of Natural History, New York, New York, USA; ANSP, Academy of Natural Sciences of Philadelphia, Pennsylvania, USA; CMN, Canadian Museum of Nature, Ottawa, Ontario, Canada; LACM, Natural History Museum of Los Angeles County, California, USA; ROM, Royal Ontario Museum, Toronto, Canada; TCM, The Children's Museum of Indianapolis, Indiana, USA; TMP, Royal Tyrrell Museum of Paleontology, Drumheller, Alberta, Canada; USNM, National Museum of Natural History, Washington, D.C., USA; YPM, Yale Peabody Museum of Natural History, New Haven, Connecticut, USA.

## Results

### Systematic Paleontology

Dinosauria Owen, 1842 [14] sensu Padian and May 1993 [15]

Ornithischia Seeley, 1887 [16] sensu Sereno 1998 [17]

Ceratopsia Marsh, 1890 [18] sensu Dodson, 1997 [19]

Ceratopsidae Marsh, 1888 [20] sensu Sereno 1998 [17]

Chasmosaurinae Lambe, 1905 [21] sensu Dodson et al., 2004 [22]

*Nedoceratops* Ukrainsky, 2007 [6]

#### Synonymy

*Diceratops* Lull vide Hatcher, 1905 [5]; *Diceratus* Mateus, 2008 [12]

#### Diagnosis

As for the type and only species.

*Nedoceratops hatcheri* Lull vide Hatcher, 1905 [5]

#### Holotype

USNM 2412, a nearly complete cranium.

#### Type Horizon and Locality

Lance Formation (late Maastrichtian) of Niobrara County, Wyoming, USA.

#### Diagnosis

Chasmosaurine ceratopsid with the following autapomorphies: nasal horncore nearly completely undifferentiated from the nasal bone; greater portion of procurved postorbital horncores forms a 90 degree angle with tooth row; and parietal fenestrae extremely small (occupying less than five percent of the total surface area of the parietal). *Nedoceratops hatcheri* is distinguished from *Triceratops* spp. in the the position of the ventral extremity of the squamosal well above the alveolar process of the maxilla, and in the presence of parietal fenestrae, which are lacking in all *Triceratops* species. *Nedoceratops hatcheri* is distinguished from *Torosaurus latus* in squamosal shape (particularly the reduced jugal notch and lack of a thickened medial margin in *N. hatcheri*), and that *N. hatcheri* has extremely reduced parietal fenestrae and a low number of episquamosals in *N. hatcheri* compared to *T. latus*.

### Description

The holotype skull for *Nedoceratops hatcheri*, USNM 2412, is nearly complete and virtually undistorted (Figures 1–3). Only minor evidence of crushing (e.g., asymmetry in preservation) is visible. Thus, the shape and orientation of cranial structures are probably largely unaltered relative to the life condition. The skull lacks only the caudal margin of the parietal and most of the right postorbital horncore, with other small absences that may have resulted from excavation, preparation or loss before burial (Figures 1B,1D,2C,3B). Nearly all of the cranial sutures are obliterated by co-ossification. This is one of several features consistent with USNM 2412 representing an “old adult” at its death.

In the following regional descriptions, orientations of cranial structures are described assuming a horizontal tooth row. Most of the palate of the skull is either unprepared or obscured by plaster and paint, and thus that region is not described in detail here. Selected measurements are presented in Table 1.

**Table 1:** Selected measurements of the holotype skull for *Nedoceratops hatcheri*, USNM 2412, in millimeters.

Measurement	
Basal skull length (rostrum to occipital condyle)	1100
Length from tip of rostrum to distal end of maxillary tooth row	804
Length from rostral rim of orbit to caudal rim of external naris	252
Length from rostral rim of orbit to tip of rostrum	854
Maximum skull width, between orbits	395
Maximum skull width, between distal tips of jugals	701
Height of snout, from bottom of premaxilla to top of nasal	414
Height of skull, from distal tip of jugal to rostral end of supratemporal fenestra	587
Nasals, width above external naris	200
Postorbital horncore, mediolateral width at base	172
Postorbital horncore, craniocaudal width at base	213
Postorbital horncore, length from top of orbit (as restored)	642
Maximum width of orbit	123
Maximum height of orbit	164
Length of jugal, from ventral margin of orbit	360
Width of jugal, at laterotemporal fenestra	157
Maximum width of occipital condyle	95
Maximum width across exoccipitals	618
Width of squamosal “blade” at widest point	464
Length of squamosal, from distal end to corner of blade (along curve)	984
Squamosal fenestra, rostrocaudal length (right side)	173
Squamosal fenestra, mediolateral width (right side)	117
Squamosal fenestra, rostrocaudal length (left side)	198
Squamosal fenestra, mediolateral width (left side)	240

All bilateral measurements were taken on the right side of the skull unless otherwise indicated.

#### Rostral and Narial Region

The nasals, premaxillae, and rostral are extensively co-ossified, so sutural relationships among these elements cannot be discerned. The dorsal margin of the “beak” (the portion of the face rostral to the nasal horncore, comprising the fused premaxillae and rostral bone) circumscribes a broad arc, with the tip of the rostral bone located just ventral to the level of the maxillary tooth row and the lowest part of the premaxilla (Figure 4). The ventral margin of the beak consists of two sinuous curves, with the rostral curve approximately twice as broad as the caudal curve (Figures 1A,1B,4), a feature best seen on the right side due to damage to the specimen (Figure 1B). Yet, compared to many other chasmosaurines (e.g., *Chasmosaurus* spp.; most examples of *Triceratops*), the degree of curvature on the beak as a whole is relatively minor.

The premaxilla displays a number of fossae and foramina consistent with the condition in other chasmosaurines (e.g., see [23]; the anatomical terminology from that paper is used here for the narial region), but plaster restoration obscures some details (Figures 1B,1D,4). The narial strut is inclined rostrally towards the dorsal end of the element, and enough original bone surface is preserved to indicate that a posterior internarial flange did not project from the caudal surface of this structure (Figure 4). A prominent triangular process extends from the ventral portion of the narial strut, and the process is situated so that its point projects dorsally into the endonaris (as seen in *Triceratops* and other closely-related chasmosaurines) rather than caudally (as seen in *Chasmosaurus* spp., *Pentaceratops sternbergi*, and others). The lateral surface of the triangular process displays a fossa, but plaster and matrix obscure any communications between this fossa and other portions of the narial complex. Similarly, the medial surfaces of the triangular processes are obscured by plaster. A small depression, the premaxillary fossa, occurs immediately rostral to the narial strut. Much of the morphology within the fossa (including a foramen piercing the fossa, as well as a strut within the fossa) is restored in plaster (Figures 1B,1D,4). A canal at the ventral edge of the fossa communicates caudally, although the extent of the canal is not fully prepared (Figure 4). The portion of the narial complex rostral to the narial strut is approximately the same maximum length as the portion caudal to the strut.

#### Nasal and Nasal Horncore

The nasal horncore of USNM 2412 (Figures 1,2,4) is one of the least pronounced for any known chasmosaurine ceratopsid skull. Rather than a discrete triangular horn (as seen in *Triceratops prorsus*; e.g., LACM 7207, YPM 1822) or knob of bone (as seen in many *Triceratops horridus*; e.g., USNM 4720, YPM 1820), the “horn” of USNM 2412 is a poorly defined swelling in the dorsal surface of the snout, similar to the condition seen in the basal centrosaurine *Diabloceratops eatoni* [24]. The snout measures approximately 150 mm in width at the midpoint of the external naris. No trace of an arcuate vessel along the rostral edge of the horncore is preserved, as seen in *Eotriceratops* and *Triceratops* [8], [25], although plaster reconstruction partly obscures the relevant region. Furthermore, extensive sutural fusion in USNM 2412 obscures any evidence of a separate epinasal ossification, if one existed in the first place. The nasal immediately caudal to the horncore is relatively thin, as seen in *Triceratops* (e.g., USNM 4720) and contrasting with the condition seen in *Coahuilaceratops magnacuerna* [26].

#### Mid-Facial Region

The dorsal surface of the mid-facial region of USNM 2412 is strongly sloped in comparison with most other chasmosaurines and much lower at its rostral end than at its caudal end when seen in lateral view (Figure 1). Only the *Triceratops horridus* skull USNM 4928 (holotype of *“Triceratops calicornis”*) approaches this morphology. The antorbital fenestra, which is caudally inclined and much longer than wide (Figure 1), is relatively small compared to the primitive condition seen in neoceratopsians (e.g., *Protoceratops*) and comparable in size to fenestrae seen in most other ceratopsids.

The general morphology of the maxilla is similar to that of *Triceratops* and *Torosaurus.* The exact number of maxillary tooth positions cannot be counted due to poor preservation, but at least 21 teeth are preserved on each side. Rostrally, no teeth are preserved, but there is space for approximately five to 10 more alveoli. The teeth are poorly preserved, so nothing can be said of the dental anatomy other than that it appears to be similar to that of other ceratopsids.

The orbits are slightly taller than wide, and a thickened antorbital buttress extends from the dorsal surface of the orbit to the rostro-ventral portion of the orbit (Figure 2). The buttress effectively occludes the orbit from visibility in rostral view of the skull (Figures 2B,2C).

The right postorbital horncore is missing only its distal quarter, but most of the left horncore is restored in plaster (see Figures 1B,1D,2C; contrary to published drawings; compare with plates 47 and 48 in [1]). The horns are remarkably erect, particularly at their bases, more so than in any undistorted *Triceratops* skull. The left horncore displays only modest rostral curvature towards its distal end, and the mid-section is moderately bowed laterally. The bases of the horncores are longer than wide.

The jugals project nearly ventrally, with only a modest lateral component and virtually no caudal inclination along the long axis of the element. The ventral margin of the jugal ends just barely below the ventral margin of the maxilla.

An epijugal is apparently present, but its sutural relations with the jugal and quadratojugal are completely obscured by fusion. Regardless, the element was not prominent even by the standards of many *Triceratops* specimens. The bone is approximately tetrahedral in shape, with the rostrodorsal surface being the longest and flattest of the three exposed surfaces. The remaining two surfaces (oriented caudally and ventrally) are comparatively rounded.

The dorsal skull roof is nearly solid, with only a small, circular frontoparietal fontanelle. The fontanelle is positioned towards the caudal half of the bases of the postorbital horncores. Prominent dorsotemporal channels pass caudally from the fontanelle, starting at a single, midline channel wider than the fontanelle itself, before passing into two narrow, caudolaterally trending channels (which then terminate in “anterior temporal foramina” [3]). This region has already been described and figured in detail elsewhere (see [27]:fig. 5 and text), so it is not treated further here.

The quadrate extends well below the ventral margin of the maxillary teeth. Its distal cotylus is divided into medial and lateral portions. The distal and caudal margin of the quadrate is clearly visible when the skull is seen in lateral view (Figure 1B), similar to the condition seen in chasmosaurines such as *Pentaceratops*, *Triceratops*, and *Utahceratops*, but contrasting with the morphology of *Chasmosaurus* and *Mojoceratops*.

The infratemporal fenestra has the shape of a rounded triangle, with the ventral margin longest, and the rostral and caudal margins approximately equal in length. The exact relationships of the elements bounding the fenestra cannot be discerned.

#### Frill

In lateral view, the parietosquamosal frill is remarkably shallow compared to the deep, saddle-shaped form seen in typical *Triceratops* specimens, particularly because the squamosal is comparatively narrow in USNM 2412 (Figure 1). The frill is erect, and the dorsal profile, where preserved, is quite straight. In rostral view, the frill is broadly arched from side to side (Figure 2B). It is shorter than basal skull length, similar to specimens of *Triceratops* and contrasting with most specimens of *Torosaurus*.

The ventral extent of the squamosal is roughly level with the ventral margin of the infratemporal fenestra and the top of the alveolar process of the maxilla (Figure 1); this contrasts with the condition typical of *Triceratops*, in which the ventral angle of the squamosal extends to the level with the maxillary tooth row or below. Some specimens of *Torosaurus latus* (e.g., ANSP 15192, MOR 1122) also show a configuration similar to USNM 2412. The squamosal's lateral margin is only gently convex, unlike the prominent crescentic profile seen in many *Triceratops* specimens (e.g., YPM 1822). Similar to *Ojoceratops fowleri*, and contrasting with *Torosaurus* spp. and most *Triceratops* spp. (MOR 004 is an exception), the ventral corner of the blade of the squamosal is indistinct. The distal end of the squamosal blade tapers to a point. Prominent bilateral fenestra pierce the rostral portion of the blade, although they are asymmetric in size and shape (Figures 1–3; Table 1). The left fenestra is approximately trapezoidal, and the squamosal in this region is greatly swollen around the fenestra's boundaries (up to 67 mm in thickness at the rostral border of the fenestra). The right squamosal fenestra is roughly oval, with the thicknesses of its margins ranging between 14 and 19 mm. These features are further described and interpreted elsewhere [13]. In contrast with previously published drawings (see [1]:plate 47, for example), the distal end of the left squamosal is reconstructed (Figure 1D).

Episquamosals are present, but the rostral episquamosals are so tightly fused to the squamosal as to be virtually indiscernible. Portions of the lateral edge of the squamosal are restored on both sides, but total episquamosal count is estimated to be five, by comparison of both left and right sides. All preserved episquamosals are approximately equal in size, at around 150 mm in length. Each episquamosal is long, low and approximately ovoid in shape (Figure 5).

Nearly the entire caudal margin of the parietal is reconstructed (Figures 1B,1D), so it is impossible to determine marginal shape or epiparietal count. Marginal epi-ossifications are restored in plaster along the squamosal-parietal contact, and there is no conclusive evidence for an epi-ossification in this position other than the plaster reconstructions (*contra* [7]). The midline bar of the parietal is completely unornamented in lateral view, with none of the midline bumps seen variably in other chasmosaurines. A small, elongate parietal fenestra occurs in the middle of the right side (133 mm long by 50 mm wide; Figure 6); the comparable region is restored on the left side of the parietal. The bone surrounding the fenestra is quite thin, between 8 and 10 mm. In caudal view, no major depressions occur on this portion of the parietal (Figure 3); the bone is uniformly flat, except for a midline depression at the rostral end of the bone (presumably for cervical musculature). Although small portions of the rostral, medial, and caudal margin are restored, the entire lateral margin of the fenestra is intact, indicating that this is a genuine feature and not simply the result of incomplete preservation. Importantly, the fenestra was explicitly noted as present by the original preparator [5].

#### Braincase

The braincase is well-preserved but only partially prepared. All of the component bones of the occipital condyle are well-fused, and no sutures are visible here. Laterally, the wing-like processes of the exoccipitals are extremely broadened (Figure 3), but no more so than is typical of *Triceratops* and other chasmosaurines (contra [5]). The ventral margins of the process extend below the occipital condyle and even the basisphenoid processes, and are partly visible from a lateral view of the skull (Figure 1). Two foramina for the exits of cranial nerves and associated structures characterize the base of the exoccipital. The contacting pterygoid bones are well-preserved and similar to those seen in *Triceratops*.

## Discussion

### Ontogenetic Status of *Nedoceratops hatcheri*

The holotype individual of *Nedoceratops hatcheri* (USNM 2412) is interpreted as an “old adult,” based on several features that are comparable with those in specimens considered to be old individuals of *Triceratops* and other ceratopsians [32], [33]. All cranial sutures are completely or nearly completely obliterated, the postorbital horncores are procurved, the bone texture on the frill is deeply vascularized and rugose rather than striated or “pebbly,” and the epiossifications are low and elongate, without a triangular peak.

### Taxonomic Status of Nedoceratops hatcheri

Opinions on the validity of *Nedoceratops hatcheri* vary greatly, although most recent work has assumed its synonymy with *Triceratops horridus* [8]–[10]. The hypothesis that *N. hatcheri* represents a transitional form between ontogenetic stages (with *Triceratops* as “young adult” and *Torosaurus* as “old adult,” [10]) is considered unlikely for multiple reasons and will be addressed below.

Synonymy of *Nedoceratops* and *Triceratops* requires either that any perceived unique features of the former are the result of pathology or developmental anomaly [2], [7] or that perceived unique features fall within the expected range of variation for *Triceratops* [2], [7], [9]. I consider these hypotheses in turn below.

Undoubtedly, some aspects of the skull of *Nedoceratops* are abnormal. For instance, the bone around the squamosal fenestra on the left side is massively thickened compared to that on the right side, and the squamosal fenestrae are asymmetric in shape. This, in conjunction with the random occurrence of squamosal fenestrae across chasmosaurines [13], indicates that the squamosal fenestrae are not reliable characters for purposes of alpha taxonomy. It is possible that the parietal fenestra is also abnormal, but the incomplete preservation of the opposite side of the frill prevents a test of this hypothesis. However, the preserved bone texture around the fenestra appears normal relative to the rest of the frill, and the rostrocaudal elongation and caudal placement of the fenestra is consistent with the condition seen in other chasmosaurines with small fenestrae (*Anchiceratops* and *Arrhinoceratops*).

Several other features on the skull clearly distinguish *Nedoceratops* from known specimens of *Triceratops*. The position of the squamosals, with the ventral border of the squamosal well above the maxillary tooth row, is unique to *N. hatcheri*. The occurrence of “anterior temporal foramina” is also unusual, shared only with some specimens of *Torosaurus latus* and *Torosaurus utahensis* [27]. The near absence of a nasal horn in *Nedoceratops* is of somewhat controversial validity. Nasal horn morphology varies greatly within *Triceratops* (related at least in part to differences between the two species [28]). However, none of the specimens known or described to date match the extreme condition observed in *N. hatcheri*. Even USNM 4720 and UCMP 128561, two specimens of *Triceratops* for which the small size of the nasal horn was used to erect new species [29], [30], have more prominent nasal ornamentation than *N. hatcheri*. Given the “old adult” status of USNM 2412 as well as the lack of an open epinasal suture or other evidence of damage, it is improbable that the nasal horn appears small due to traumatic or taphonomic loss of the epinasal ossification (contra [2], [3]).

The preponderance of unusual features seen in the type and only skull of *Nedoceratops hatcheri* may either be explained as a whole suite of abnormalities in a single aberrant individual of *Triceratops*, or be explained as a suite of autapomorphies characterizing a taxon distinct from *Triceratops*. I consider the latter explanation to be most likely. This hypothesis could be further bolstered by the discovery and description of additional specimens with similar morphology, or refuted by the identification of undisputed specimens of *Triceratops* that overlap in all aspects of morphology with *N. hatcheri* or preserve a mélange of character-states that are intermediate between known *Triceratops* specimens and *Nedoceratops*. Additional information relevant to the latter case is presented below and in [10].

Assuming that it is a separate taxon, *N. hatcheri* is closely allied with the clade including *Triceratops* and *Torosaurus*. *Nedoceratops* shares several features with *Triceratops* spp. to the exclusion of the coeval *Torosaurus latus*, including a comparatively short frill relative to skull length, low number of episquamosals, and a lack of a thickened medial margin or prominent concave depression on the dorsal surface of the squamosal. A recent phylogenetic analysis of chasmosaurines [31] recovers *N. hatcheri* as sister taxon to *Triceratops* spp., with a decay index of 1 separating the two clades. These taxa are united by a single unambigous synapomorphy, a short parietosquamosal frill (see text S1 in [31]).

### Taxonomy of Late Maastrichtian Chasmosaurines

Scannella and Horner [10] proposed that the large chasmosaurine *Torosaurus latus* was synonymous with coeval *Triceratops* spp. Although *T. latus* differs from *Triceratops* in features such as frill fenestration, frill thickness, and number of epiossifications, it was suggested that these differences were manifested during ontogeny of a single taxon. In other words, *Triceratops* ontogenetically transformed into *Torosaurus* as a fully-grown adult. The holotype skull of *Nedoceratops hatcheri*, USNM 2412, with its small parietal fenestrae and thin frill, was cited as a possible transitional stage between the “young adult” and “old adult” conditions. This hypothesis (here referred to as the “Ontogenetic Trajectory Hypothesis,” or OTH) has major implications for our understanding of ceratopsid evolution and diversity at the Cretaceous-Paleocene boundary. In the section below, the names *Triceratops* and *Torosaurus* are used in their traditional sense, as if they were separate taxa.

One assumption of the OTH is that the number of epiossifications on the ceratopsid frill increased through ontogeny. Typical *Triceratops* spp. frills have between five (e.g., TCM 2001.93.1) and seven (e.g., MOR 1120) episquamosals or undulations for placement of episquamosals, and five or six epiparietals or spaces for epiparietals [10], not counting the ossification spanning the squamosal-parietal suture. In contrast, *Torosaurus latus* consistently has seven episquamosals and between 10 and 12 epiparietals [34]. Thus, the OTH would require the typical addition of between zero and two episquamosals and between five and seven epiparietals during ontogeny, along with the loss of the midline epiparietal that characterizes nearly all specimens of *Triceratops* but is absent in all known *Torosaurus*.

None of the known ontogenetic series for any other ceratopsid suggest that such a large addition of epiossifications ever happened. For instance, the frill of TMP 82.16.11, which likely belongs to a juvenile *Centrosaurus apertus* approximately one-fifth of adult size, has four scallops on each squamosal and an estimated twelve scallops on the parietal [35]. Assuming that each scallop corresponded to an epiossification, this is within the range of variation for the count seen in full-sized, adult specimens of *C. apertus* (four [e.g., ROM 767] or five [e.g., TMP 65.12.2] episquamosals and 12 [e.g., CMN 8798] or 14 [e.g., AMNH 5239] epiparietals; personal observation). Thus, *C. apertus* probably did not add a large number of epiossifications, if any, during ontogeny. In the event that TMP 82.16.11 matured into an individual with five episquamosals and 14 epiparietals, it would have added no more than one or two epiossifications to each of these elements during its entire growth sequence. Similar patterns of constant or near-constant epiossification counts apparently occurred in other centrosaurines for which at least partial ontogenetic series are known (e.g., *Styracosaurus albertensis*; *Einiosaurus procurvicornis*).

Although ontogenetic series for the frill are poorly known in chasmosaurine ceratopsids outside of *Triceratops*, important information is offered by a juvenile squamosal, TMP 98.123.1, from the Dinosaur Park Formation of Alberta, assignable to either *Chasmosaurus* sp. or *Mojoceratops perifania*. The element measures 200 mm from the jugal notch to the distal end (compared with a measurement of 910 mm in ROM 839, a presumed adult specimen referable to *Chasmosaurus belli*; [36]), and has 10 marginal undulations. Presumed adult specimens of *Chasmosaurus* spp. and *Mojoceratops perifania* have between 6 and 10 episquamosals or marginal undulations [36]. Thus, there is little evidence that any chasmosaurine consistently added marginal ossifications during ontogeny, and any differences in count between specimens can be attributed to specific differences or individual variation (note that Godfrey and Holmes [36] suggested the number of undulations increased ontogenetically, an interpretation not supported here). Even squamosals from “baby” and juvenile *Triceratops* have between five and seven scallops for attachment of episquamosals [37] (see also figure 3 in [10]), corresponding precisely to the number found in most adult-sized individuals. The smallest known parietal of *Triceratops* has five places for epiparietals (not counting the ossification spanning the parietal-squamosal suture; [10]), congruent with the count seen in many adult specimens five times its size. The only possible exception may be in *Agujaceratops mariscalensis*, in which two presumed juvenile or subadult specimens have six episquamosals [38] and a single known presumed adult has ten [39]. Given the variation within *Chasmosaurus* spp. and the small sample size for *A. mariscalensis*, this pattern is just as likely the result of individual variation as it is the result of ontogenetic changes.

The OTH also requires that *Triceratops* acquire parietal fenestrae at the very end of ontogeny. This would contrast sharply with the known conditions in *Protoceratops andrewsi* [40] and *Centrosaurus apertus* [35], in which parietal fenestrae appear at a very early ontogenetic stage. Scannella and Horner [10] proposed that the acquisition of fenestrae in *Triceratops* was associated with a “striated” surface texture at the caudal end of the parietal and a “pebbly” surface texture at the future site of the fenestrae. Simultaneously, the ventral surface of the parietal developed shallow depressions which later transformed into full fenestrae. Specimens of both *Triceratops* (AMNH 5116; [10]:fig. 5C) and *Torosaurus* (MOR 981; [10]:fig. 5D, presumably early within the transition) do indeed show this bone texture. Thus, assuming that the *Nedoceratops hatcheri* holotype skull, USNM 2412, is transitional between the two morphs, both the striated/pebbly bone texture as well as ventral depressions should be visible on the parietal in this specimen. Yet, USNM 2412 instead shows well-developed neurovascular impressions rather than striated or pebbly bone across the entire preserved ventral and dorsal surfaces of the frill (Figures 5,6), and no depression occurs around the preserved parietal fenestra (although the parietal is very thin in this area). It is far more likely that the depressions on the ventral surface of the parietal observed in some specimens of *Triceratops* correspond to insertion areas for cervical musculature [41]. Thus, the “incipient fenestra” in MOR 2946 ([10]:fig. 1C) may instead represent an area for muscle attachment.

Although bone resorption (associated with a “mottled” or “pebbly” texture; [10], [42], [43]) occurs on both the dorsal and ventral surfaces of the frill in *Triceratops*, this texture is not unambiguously associated with formation of fenestrae in other ceratopsids. In fact, it is probably a general feature of cranial bone growth in the clade. For instance, this texture also occurs on the squamosals of *Centrosaurus*, elements which are not normally fenestrated [43], as well as on the midline of the parietal [42]. Furthermore, ontogenetic evidence for *Centrosaurus* strongly suggests that cranial bone (particularly in the frill) passed through three sequential ontogenetic stages: 1) long-grained texture; 2) mottled texture; and 3) adult texture (characterized by a rugose surface occasionally with neurovascular impressions; [32], [42]). Assuming that this is the case in *Triceratops* (based on the fact that cranial elements from obvious juveniles lack the adult bone texture), and assuming the OTH is correct, finding definitive *Triceratops* skulls with “adult” bone texture across the entire parietal should not be likely. Yet, numerous examples of this condition exist (e.g., USNM 2100, YPM 1822). The fact that a specimen of *Torosaurus latus*, MOR 1122, shows the most mature bone texture in a histological sample of five individuals [10] is intriguing, but the published sample size is too small to confirm that this is a consistent histological feature of the morphotype or that “full adult” *Triceratops* lack this texture. Additional histological sampling is needed to address this question.

In sum, the OTH requires that *Triceratops* underwent a sequence of ontogenetic changes that was completely unique among ceratopsids. Addition of numerous epiossifications, acquisition of parietal fenestrae at a very late ontogenetic stage, and reversion of adult bone texture to mottled bone texture and finally a return to adult texture during ontogeny are unlikely (although certainly not impossible). Furthermore, the *Nedoceratops hatcheri* skull USNM 2412 does not present an intermediate ontogenetic step between *Triceratops* and *Torosaurus* morphotypes, particularly based on the rugose surface texture on the parietal in this specimen. A perceived lack of juvenile *Torosaurus* specimens may simply reflect the fact that this taxon is quite rare, rather than that such specimens only occur as *Triceratops*. Indeed, baby and juvenile specimens (sensu [33]) of *Triceratops* (which is otherwise quite common in the Hell Creek Formation) were virtually unknown and unrecognized until recently [44]. YPM 1831, a partial skull, is one possible candidate for a subadult *Torosaurus latus*. This specimen displays a number of subadult characteristics, including an epinasal that is not fused with the underlying nasals, an epijugal that is not fused to the jugal, and open sutures between the exoccipitals and basioccipital of the occipital condyle [1]. Furthermore, the bone on the surface of the frill is smooth (consistent with subadult status) and no epiossifications are readily visible (suggesting that they may have been disarticulated prior to burial). Thus, it is far more likely that *Triceratops*, *Torosaurus*, and probably *Nedoceratops*, are distinct taxa.

### Conclusions

Despite its convoluted taxonomic history, *Nedoceratops hatcheri* does indeed display several features that distinguish it from typical *Triceratops* and *Torosaurus* specimens, as well as other chasmosaurines (such as the profile of the squamosal, lack of a nasal horn, and presence of small parietal fenestrae). Even if *N. hatcheri* represents an aberrant *Triceratops*, the anatomy of *N. hatcheri* is inconsistent with the hypothesis that it is a transitional form between the “young adult” (classic *Triceratops*) and “old adult” (classic *Torosaurus*) morphotypes of a single taxon. Unless *Triceratops* underwent ontogenetic changes radically different from any other known ceratopsid, it seems most likely that the latter two taxa are also distinct from each other.

In a broader context, the number of valid ceratopsid species living in North America during the latest Cretaceous is of considerable interest for interpreting changes in dinosaur diversity (if any) just prior to their extinction at the Cretaceous-Paleocene boundary. Assuming that *Triceratops*, *Torosaurus*, *Nedoceratops*, and possibly *Tatankaceratops* are all valid taxa, raw species richness for chasmosaurines in the northern Western Interior of North America during the late Maastrichtian meets or exceeds that for the late Campanian of Alberta. Yet, by sheer number of specimens, *Triceratops* is clearly most common in the collected sample. The rarity of other chasmosaurines may reflect a true regional predominance of *Triceratops*, local rarity of non-*Triceratops* in the Hell Creek and Lance Formations due to ecological factors (i.e., other taxa were more common elsewhere), or a taphonomic artifact.

## Materials and Methods

The holotype skull of *Diceratops hatcheri*, USNM 2412, was studied first-hand on three different occasions in order to map areas of reconstruction, verify morphology, and obtain photographs. Measurements were recorded to the nearest millimeter using a cloth measuring tape and digital calipers.
